# Single-center experience of simultaneous bilateral uni-portal video-assisted thoracoscopic surgery for multiple ground-glass opacities

**DOI:** 10.1186/s13019-020-01107-0

**Published:** 2020-04-23

**Authors:** Rirong Qu, Zhipeng Hao, Yang Zhang, Lei Bie, Xiangning Fu, Ni Zhang

**Affiliations:** 1 Department of Thoracic Surgery, Tongji Hospital, Tongji Medical College, Huazhong University of Science and Technology, Jiefang Street1095, Wuhan, 430030 Huebei provience China; 2 Department of Obstetrics and Gynecology, Tongji Hospital, Tongji Medical College, Huazhong University of Science and Technology, Jiefang Street1095, Wuhan, 430030 Huebei provience China

**Keywords:** Multiple ground-glass opacity (GGO);simultaneous;bilateral;video-assisted thoracoscopic surgery (VATS)

## Abstract

**Background:**

There is an increasing incidence rate of ground-glass opacity (GGO), especially for multiple GGOs (≥2). Whether it is safe and feasible to have bilateral simultaneous surgical resection remains unknown. The purpose of this study is to summarize the experience of surgical resection of patients with multiple GGOs in our Hospital in recent years, and to discuss the above questions.

**Methods:**

Clinical datas of patients who underwent one-stage bilateral uni-portal VATS resections of multiple pulmonary ground glass opacities and had routine pathological examination were collected from May 2016 to May 2019 in our hospital.

**Results:**

A total of 34 patients underwent simultaneous bilateral surgical resection of multiple GGO lesions, 28 were women,6 were men, the average age of total patients was 57.9 ± 6.7 years. All patients underwent bilateral uni-portal video-assisted thoracoscopic surgery (Uni-portal VATS), the average intraoperative blood loss was 100.9 ± 67.7 ml, the average operation time was 140 ± 74.8 min, the average thoracic drainage time was 2.8 ± 3.1 days, and the average postoperative hospital stay was 4.2 ± 4.3 days. Postoperative complications including: 2 cases of infection, 3 cases of atrial fibrillation, and 5 cases of persistent air leakage for more than 3 days. All of them improved after treatment, and there were no serious complications and deaths in perioperative period. A total of 76 GGO lesions were resected, with a total malignancy rate of 81.6%, including 40 were pure GGO, of which 28 were malignant (70%), and the average diameter of them were 9.6 ± 3.8 mm; 36 were mixed GGO, of which 34 were malignant (94.4%), the average diameter of them were 15.6 ± 6.6 mm.Mean postoperative follow-up was 28.4 (range, 3–39) months. There was neither recurrence nor deaths at final follow-up.

**Conclusion:**

The malignancy rate of multiple GGOs is high. Therefore, when the lung function is allowed,one-stage bilateral uni-portal VATS can be considered. According to experience of main surgeon and the frozen biopsy, either sub-lobar resection or lobectomy was acceptable. The risk of postoperative complications and the prognosis were optimal.

## Background

A ground glass opacity (GGO) is defined as a hazy opacity that does not obscure the view of underlying bronchial structures or pulmonary vessels on HRCT [1]. According to whether it contains solid components, it is divided into pure ground glass (pGGO) and mixed ground-glass opacity (mGGO). When the number of ground glass in the lungs of the patient is more than two, it is called multiple GGOs [2, 3]. The clinical features of the ground glass opacity are extremely diverse,including malignancies and benign conditions,for example,focal interstitial fibrosis, inflammation, haemorrhage and adenocarcinoma [4, 5]. However, in the CT manifestation [6, 7], early lung adenocarcinoma is mostly characterized by pulmonary ground glass opacity. In recent years, With the development of diagnostic techniques, especially the wide application of high-resolution computed tomography (HRCT) and PET-CT,and the increasing health awareness of people, the detection rate of ground-glass opacity (GGO) is increasing in recent years [4, 8, 9]. There is some controversy about how to deal with this part of patients, especially those with imaging diagnosis of bilateral early stage lung cancer. Therefore, our center summarized the experience of simultaneous bilateral surgical resection of multiple GGO patients from May 2016 to May 2019, and conducted a preliminary discussion on the above issues.

## Methods

This retrospective study was approved by the Ethics Committee of Huazhong University of Science and Technology,Tongji Medical College, that also waived the requirement of informed consent for the use of the patient’s medical data.

### Patients

From May 2016 to May 2019,the data of 34 patients who underwent simultaneous bilateral uni-portal VATS multiple GGO resections were retrospectively analyzed in the Department of Thoracic Surgery,Wuhan Tongji Hospital. Of the 34 patients studied,28 were women,6 were men, the average age of total patients was 57.9 ± 6.7 years (range,41–69 years). All patients were in fine physical condition and they have no history of tumors. 76 GGO lesions were identified in the 34 patients,and all patients underwent preoperative examinations to exclude distant metastases. Preoperative examinations include chest enhanced CT, head CT or head MRI, bone scans, and abdominal ultrasound, PET-CT is performed if necessary. Patients who had anti-inflammatory treatment for at least 1 week before surgery, when conservative treatment is ineffective and no benign outcomes are observed, surgical treatment should be considered. In all patients, we try to obtain a pathological diagnosis through preoperative percutaneous lung biopsy or frozen sections during operation to guide the extent of resection. The decision of resection of multiple GGO was made after discussing with the thoracic surgeons, oncologists, and radiologists. The clinical characteristics of patients are shown in Table 1.

**Table 1:** Clinical characteristics of Patient

Variables	N(%)	Mean value
Sex		
Male	6 (17.6)	
Female	28 (82.4)	
Age (years)		57.9 ± 6.7
≥ 60	16 (47.1)	
<60	18 (52.9)	
Number of lesions		
2	28 (82.4)	
>2	6 (17.6)	
Type of lesions		
pGGO	40 (52.6)	
mGGO	36 (47.4)	
Combined underlying disease		
Yes	6 (17.6)	
No	28 (82.4)	
Smoking history		
Yes	6 (17.6)	
No	28 (82.4)	
Family history of cancer		
Yes	7 (20.5)	
No	27 (79.5)	
Ejection fraction^a^		
≥ 60	14 (41.2)	60.9 ± 1.4
55–59	20 (58.8)	
p-FEV1%^b^		
≥ 100	10 (29.4)	95 ± 15.8
80–100	14 (41.2)	
≤ 80	10 (29.4)	

a, EF is the percentage of left ventricular blood volume pumped by the heart in a single contraction;b, p-FEV1%: percent of the predicted FEV1;

### Surgical procedures

**Fig. 1:**
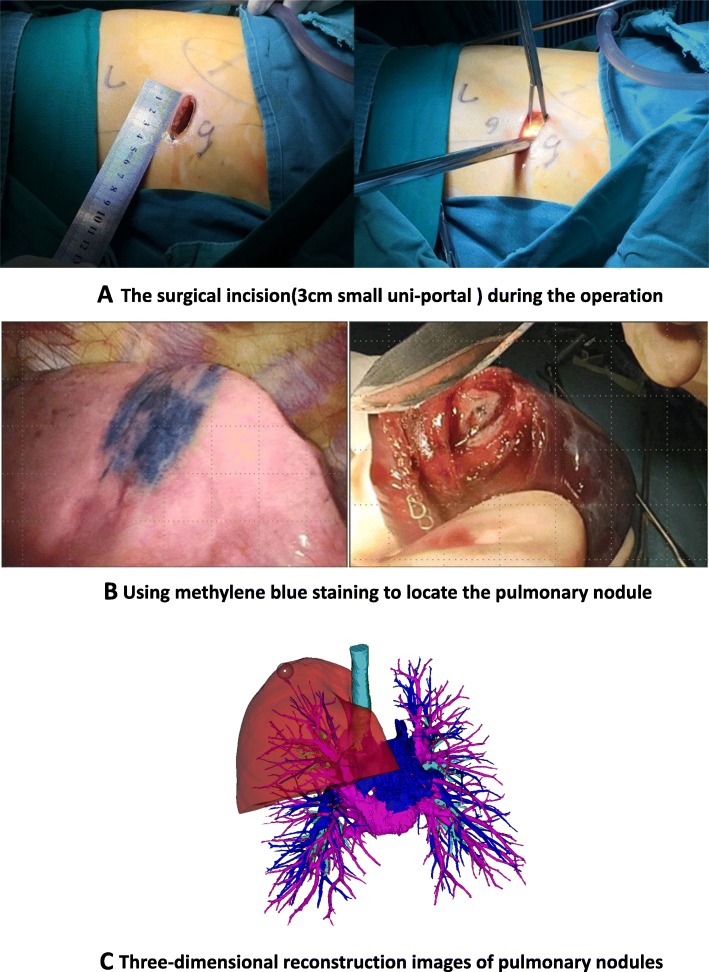
**a** The surgical incision (3 cm small uni-portal) during the operation. **b** Using methylene blue staining to locate the pulmonary nodules. **c** Three-dimensional reconstruction images of pulmonary nodules

All procedures were performed with intravenous inhalation combined with anesthesia + double lumen endotracheal intubation. The operation used 3 cm small uni-portal method **(**Figure 1**a)**: the patient’s lateral side of the midline of the 5th intercostal line 3 cm incision into the thoracoscope (the left side can also choose the 6th intercostal space), elbow cavity mirror suction device, electrocoagulation hook, If necessary, insert a double joint clamp to hold the lobes. At the end of the operation, two 12G microtubules were placed for chest drainage. After the end of one side of the operation, turn over the same side of the same the law. The specific surgical plan is based on the size and location of the bilateral lung GGO, the lung function reserve and the intraoperative frozen examination results. All patients underwent preoperative three-dimensional CT reconstruction and CT-guided methylene blue staining location except central lensions **(**Fig. 1b and Fig. 1c). In order to avoid the spread of methylene blue, we take the following methods: 1. Surgery as soon as possible after staining is complete; 2. The place marked by methylene blue is next to the lesion, and it will not affect the pathological diagnosis of the lesion. Surgical strategy: ①Pure GGO preferentially choose wedge resection or segmentectomy. ② Peripheral lesions are preferentially treated with wedge resection, and if they are central lesions, lobectomy is performed. ③The lesion is larger than 2 cm and the imaging is considered as an invasive lesion, which will be considered lobectomy. ④Rapid pathology during resection of the lesion is considered as lobectomy for invasive adenocarcinoma.⑤The priority side of the operation is based on the preoperative three-dimensional reconstruction to select the side of the lung tissue that is expected to be resected, such as the wedge resection or segmentectomy. If all are sublobar resection, the right side surgery is preferred.

### Follow-up

All patients with pathologically-proven cancers were followed up after surgery. Follow-up was performed by outpatient or telephone follow-up. The follow-up time was calculated from the day after surgery and was followed up until August 2019. In the first year after surgery, chest CT, tumor markers and abdominal ultrasound were reviewed every 3 months; in the second year after surgery, the above indicators were reviewed every 6 months; the above indicators were reviewed annually.

### Statistical analysis

Statistical analysis was performed using SPSS 23.0 software. The normal distribution of the measurement data was described by mean ± standard deviation (^−^X ± S); the count data was described by percentage (%).

## Results

### Perioperative results of patients

All patients underwent bilateral single-portal thoracoscopic surgery. The operation was successfully completed without transposition of the thoracotomy. Among them, 8 patients underwent systemic lymph node dissection, 10 patients underwent lymph node sampling, and the remaining patients did not undergo lymph node dissection according to preoperative pathological results, intraoperative frozen pathology and lesion size. No lymph node metastasis was found after postoperative pathology. The mean intraoperative blood loss was 100.9 ± 67.7 ml, the average operation time was 140 ± 74.8 min, the average thoracic drainage time was 2.8 ± 3.1 days, the average postoperative hospital duration was 4.2 ± 4.3 days, and the postoperative complications included 2 cases of pulmonary infection, 3 cases of atrial fibrillation, and persistent air leakage for more than 3 days was observed in 5 cases. After treatment, they all improved. No severe perioperative complications or deaths occurred. All patients were successfully discharged.

### Clinical characteristics of the GGOs

A total of 76 GGO lesions were removed from 34 patients, including 40 pGGO, 36 mGGO. 34 were in the left upper lung, 22 were in the right upper lung, 6 were in the right middle lung, 8 were in the right lower lung, and 6 were in the lower left lung. There were 46 peripheral lesions and 30 central lesions. There were 40 lesions with a diameter of ≥10 mm and 36 lesions with a diameter of <10 mm. All patients underwent surgical resection in 6 cases of bilateral wedge resection, 16 cases of segmental wedge resection, 2 cases of segmental segmental resection, 8 cases of lobectomy plus wedge resection, and 2 cases of lobectomy combined with segmental resection. In the time interval between the first time finding and the surgery, there were 20 lesions with different degrees of growth, of which 75% were mGGO. Clinical characteristics of the GGOs are shown in Table 2.

**Table 2:** Clinical characteristics of GGO

Varibles	N(%)
GGO pattern	
pGGO	40 (52.6)
mGGO	36 (47.4)
GGO size	
≥ 10 mm	40 (52.6)
<10 mm	36 (47.4)
Surgical procedures	
Wedge resection-wedge resection (W/W)	6 (17.6)
Segmentectomy-wedge resection(S/W)	16 (47.1)
Segmentectomy-segmentectomy(S/S)	2 (5.9)
Lobar-wedge resection(L/W)	8 (23.5)
Lobar-segmentectomy(L/S)	2 (5.9)
Lobar-lobar(L/L)	0 (0)
GGO location	
Central	30 (39.5)
Peripheral	46 (60.5)
Occupying lobe	
RUL	22 (28.9)
RML	6 (7.9)
RLL	8 (10.5)
LUL	34 (44.8)
LLL	6 (7.9)
**Pathological residue**	
**R0**	**75 (98.7)**
**R1**	**1 (1.3)**
**R2**	**0 (0)**
Growth after first discovered	
Yes	20 (26.3)
No	56 (73.7)
Next generation sequencing	
EGFR mutation	20 (26.3)
WT	35 (46.1)
No texting	21 (27.6)

### Pathological characteristics and EGFR gene analysis of the GGOs

A total of 76 GGO lesions were resected, with a total malignancy rate of 81.6%, including 40 pure GGO, of which 28 were malignant (70%), and the average diameter of them were 9.6 ± 3.8 mm; 36 were mixed GGO, of which 34 were malignant (94.4%), the average diameter of them were 15.6 ± 6.6 mm. The results of pathological examination of 76 GGOs revealed 20 AIS (26.3%), 14 MIA (18.4%), 20 IA (26.3%), 8 AAH (10.5%),and 14 other benign nodules (18.4%).Among them, pGGO was mainly in situ adenocarcinoma (AIS), with 14 (35%); mGGO was mainly invasive adenocarcinoma (IA), with 16 (44.4%). The lesions that were growed during the follow-up were observed before surgery, and the postoperative pathology was all malignant (Table 3). Most of the lesions with malignant pathology were genetically tested. A few of them were not detected due to insufficient tissue specimens and personal reasons. Among them, there were 20 lesions with EGFR mutation, mainly L858R and 19Del mutations, and 35 lesions without EGFR mutation (Table 2). Compared with the rapid frozen pathology, there were 12 lesions with pathological upgrade, and 3 of them were upgraded to invasive adenocarcinoma, resulting one patient had a second operation due to insufficient resection**(R1)**.

**Table 3:** Pathological analysis of GGO

	The rapid pathology					Postoperative routine pathology					Mean diameter (mm)	
	Benign	AAH	AIS	MIA	IA	Benign	AAH	AIS	MIA	IA	Benign	Malignant
pGGO	18	6	4	2	2	12	4	14	6	4	8.3 ± 3.3	9.6 ± 3.8
mGGO	4	8	8	2	10	2	4	6	8	16	8.5 ± 3.7	15.6 ± 6.6
Total	22	14	12	4	12	14	8	20	14	20	8.3 ± 3.1	15.3 ± 9.7

### Follow-up and survival

Mean postoperative follow-up in cases of primary lung cancer was 28.4 (range, 3–39) months. There was neither recurrence nor deaths at final follow-up.

## Discussion

With the development of imaging technology and the improvement of the living standards of our people and the awareness of physical examination, the detection rate of multiple nodules in both lungs has been rising, especially multiple GGO. At present, there is no consensus on the diagnosis and treatment of multiple GGO in both lungs. In terms of imaging findings, lung GGO is more of an early stage lung adenocarcinoma, which should be detected early, diagnosed early, and treated early. Surgery is currently the best treatment for early stage lung cancer. Lino and Colleague’s study [10] proved that the effect of one-stage bilateral surgery for bilateral lung cancer is better than one side surgery combined with contralateral radiotherapy or combination chemotherapy. However, for GGO, which is considered to be malignant in both lungs, it is controversial to take one-stage surgery or two-stage surgery. One-Stage surgery has relatively small surgical trauma and a low incidence of postoperative complications, which seems to be safer, but considering that patients need to wait for about 1 month to perform a second operation and may lead to tumor progression on the other side during the waiting process. This will greatly lead to anxiety and affect their life. The study also reported [11] that the trauma of the first-stage surgery, such as the release of inflammatory factors and the destruction of the immune system, may increase the risk of secondary surgery. Although the trauma of one-stage operation is large and the need to remove more lung tissue, this may increase the potential perioperative risk, but its advantages are also obvious, it can solve the bilateral lesions in one operation, reduce the pain of the patient’s secondary surgery, save medical resources, and more importantly, it can reduce the risk of tumor progression caused by staged surgery. Several studies [12–14] have shown that simultaneous bilateral VATS is safe and feasible, and does not increase the risk of perioperative surgery. In our study, there were no serious complications and deaths in perioperative period, all patients were discharged smoothly, except for the average operation time compared with unilateral surgery, intraoperative blood loss, postoperative thoracic drainage time and postoperative hospital stay days did not increase significantly, and the results were satisfactory. This indicates that one-stage bilateral uni-portal VATS for multiple GGO is safe and feasible. There are some reasons: First, accurate location of the lesion before surgery, we use CT-guided methylene blue staining location and preoperative three-dimensional reconstruction technology to double positioning of GGO lesions, which helps us to develop the best operation plan before surgery In order to avoid excessive removal of normal lung tissue. Second, the use of single-hole thoracoscopic techniques has greatly reduced the trauma of the patients during surgery, the postoperative pain is significantly reduced, and the recovery is accelerated. Third, we routinely use painkillers and analgesia pumps after surgery, which help patients get out of bed early, carry out effective cough and expectoration, and promote rapid recovery after surgery.

However, there is no consensus on the specific methods of surgery for GGOs, but it is generally considered that sublobar resection is more appropriate than lobectomy. Miller and colleagues [14] found that lobectomy, sublobar resection (segmentectomy and wedge resection) for ≤10 mm tumors were compared, there was no statistical difference in survival rate and local recurrence rate. Lee and colleagues [15] suggested that the surgical approach of pGGO with pathological types of AIS and MIA has been recommended for sublobar resection rather than lobectomy. There are also reports [11, 16, 17] in the literature that multiple GGO patients with surgical resection,the prognosis is satisfactory, even sub-lobectomy does not affect the prognosis. In view of the good prognosis of multiple GGOs [18–20], we suggest that sub-lobectomy should be performed as much as possible for patients with multiple GGOs in the same period of surgery, which can ensure the prognosis and make the patient better quality of life. However, according to the rapid pathological results of the operation, the best resection plan was adopted. In this study, there were 12 lesions with pathological upgrade, of which 3 were invasive adenocarcinoma, resulting one patient had a second operation due to insufficient resection. So sometimes we can not rely entirely on intraoperative rapid pathology, but should make a more reasonable choice of resection according to the size of the patient’s lesions, the ratio of solid components and imaging findings.

For the multiple GGO in both lungs, the current controversy is whether all GGO should be removed simultaneously. Shimada and colleagues [16] suggested that the superior lesion should be resected. After the main lesion is removed, whether the remaining GGO lesion continues to grow, or a new GGO lesion appears, or the remaining GGO lesion is not treated, it will not affect prognosis. Our study showed that a total malignancy rate was 81.6%, including 40 pure GGO, of which 28 were malignant (70%), and the average diameter of them were 9.6 ± 3.8 mm; 36 were mixed GGO, of which 34 were malignant (94.4%), the average diameter of them were 15.6 ± 6.6 mm. This indicates that there is a high possibility that multiple GGOs are malignant lesions, especially those that have growed or not disappeared during follow-up. When lung function is allowed, single-stage resection of multiple pulmonary ground glass opacities can be considered. If the patient is unable to tolerate it, mGGO and pGGO lesions with a diameter greater than 9.6 mm should be given priority for surgical resection. For the remaining GGO lesions, regular follow-up can be selected, once the solid component of the lesion increases or the volume increases, reoperation should be considered.

For the multiple GGO in both lungs, it is necessary to perform routine genetic testing for pathologically malignant lesions. Some researchers [21] believe that multiple GGO should be considered as multiple primary lung adenocarcinoma, rather than intrapulmonary metastasis. Although most primary adenocarcinomas can be distinguished from intrapulmonary metastases by clinical manifestations, imaging and morphological features, genetic testing can provide patients with more accurate histological typing and pathological staging [22]. In this study, 55 of the 76 GGO lesions were detected by EGFR, and 25 of them had gene mutations. Among them, 4 patients had different types of mutations in the malignant lesions of both lungs, which were consistent with the characteristics of multiple primary lung adenocarcinoma. Genetic testing can not only identify intrapulmonary metastases, but more importantly, provide a basis for targeted therapy in patients with tumor recurrence in the future.

However, our study had some limitations and shortcomings. The first and a major limitation of this study is its retrospective nature, thus, the selected bias definitely existed. Second, there was no control group in this study. Therefore, we could not compare this method to two-stage surgery for multiple lung nodules. Third, the period of follow-up was not long. Although no patient developed new lung nodules or distant metastasis, further follow-up results are definitely required. Last,the sample size of this study is small, and it needs to be confirmed by prospective and large sample studies in the future.

## Conclusion

In summary, Our results suggest that the lesions of patients with multiple GGO are highly malignant, when lung function is allowed, one-stage bilateral uni-portal VATS can be considered. Simultaneous bilateral uni-portal VATS is feasible and safe. We believe that with the development of thoracoscopic techniques and the application of postoperative rapid recovery concept, simultaneous bilateral uni-portal VATS will give patients a greater benefit.

## Data Availability

All data generated or analyzed during this study are included in this article.

## Abbreviations

GGO: Ground-glass opacity
VATS: Video-assisted thoracoscopic surgery
HRCT: High-resolution computed tomography
CT: Computed tomography
PET-CT: Positron emission tomography-computed tomography
AAH: Atypical adenocarcinoma hyperplasia
AIS: Adenocarcinoma in situ
MIA: Minimally invasive adenocarcinoma
IA: Invasive adenocarcinoma
EGFR: Epidermal growth factor receptor
